# An Improved Procedure for Subcellular Spatial Alignment during Live-Cell CLEM

**DOI:** 10.1371/journal.pone.0095967

**Published:** 2014-04-22

**Authors:** Benjamin S. Padman, Markus Bach, Georg Ramm

**Affiliations:** 1 Department of Biochemistry and Molecular Biology, Monash University, Clayton campus, Victoria, Australia; 2 Monash Micro Imaging, Monash University, Clayton campus, Victoria, Australia; The University of Queensland, Australia

## Abstract

Live-cell correlative light and electron microscopy (CLEM) offers unique insights into the ultrastructure of dynamic cellular processes. A critical and technically challenging part of CLEM is the 3-dimensional relocation of the intracellular region of interest during sample processing. We have developed a simple CLEM procedure that uses toner particles from a laser printer as orientation marks. This facilitates easy tracking of a region of interest even by eye throughout the whole procedure. Combined with subcellular fluorescence markers for the plasma membrane and nucleus, the toner particles allow for precise subcellular spatial alignment of the optical and electron microscopy data sets. The toner-based reference grid is printed and transferred onto a polymer film using a standard office printer and laminator. We have also designed a polymer film holder that is compatible with most inverted microscopes, and have validated our strategy by following the ultrastructure of mitochondria that were selectively photo-irradiated during live-cell microscopy. In summary, our inexpensive and robust CLEM procedure simplifies optical imaging, without limiting the choice of optical microscope.

## Introduction

Correlative light and electron microscopy (CLEM) combines the advantages of Electron Microscopy (EM) and Light Microscopy (LM) by spatially aligning imaging data sets of the same region of interest. CLEM has proven to be a powerful technique for investigating rare and dynamic events at an ultrastructural level, providing unprecedented insights into membrane trafficking [1], [2], neurophysiology [3]–[6], and chromosome dynamics [7], [8].

The primary challenge of CLEM sample preparation is the relocation of a target identified by optical microscopy. Normally, this is aided by marking the sample with a reference grid either by attaching a TEM finder grid to the sample [9], depositing a layer of gold [10] or carbon [11] through a TEM finder grid, etching the sample surface with a needle or focussed ion beam [12], or using commercially available photo-etched glass coverslips [2]. Limitations of these grid preparation strategies include poor visibility, restrictions to the practical imaging area and/or reliance on specialised equipment.

CLEM of living cells presents additional challenges as cells have to be imaged *in situ* on a substrate compatible with cell culture, optical imaging and EM sample processing. Photoetched gridded-glass coverslips have been used successfully for CLEM including in combination with the Tokuyasu technique [13]. Polymer films such as Aclar are increasingly used for CLEM [14]–[19] and provide additional advantages as they are easier to handle during sample processing [20], and are compatible with cryopreservation by High Pressure Freezing (HPF) [21].

However, pliable polymer films require a glass coverslip for support during optical imaging [9], [22], which reduces optical image quality by increasing the number of refractive interfaces [23].

We sought to develop a simple and robust workflow by optimizing the use of polymer film substrates for live-cell CLEM. Importantly, our procedure uses the polymer film directly as a substrate for optical imaging. This eliminates the requirement for a supporting glass coverslip and is achieved by a purpose designed film holder that can be used in combination with any polymer film. The film holder is compatible with most inverted microscopes. We compare the suitability of three different polymer films for optical imaging, cell culture, and sample preparation to identify an optimal polymer substrate for CLEM imaging. In addition, we present a novel technique for preparing high-contrast customizable reference grids on CLEM substrates using conventional office equipment, based on the “toner transfer” technique [24], [25]. In summary, our procedure provides a simple and robust CLEM workflow which can be established in a standard EM laboratory.

## Results

### A universal film holder for CLEM

**Figure 1:**
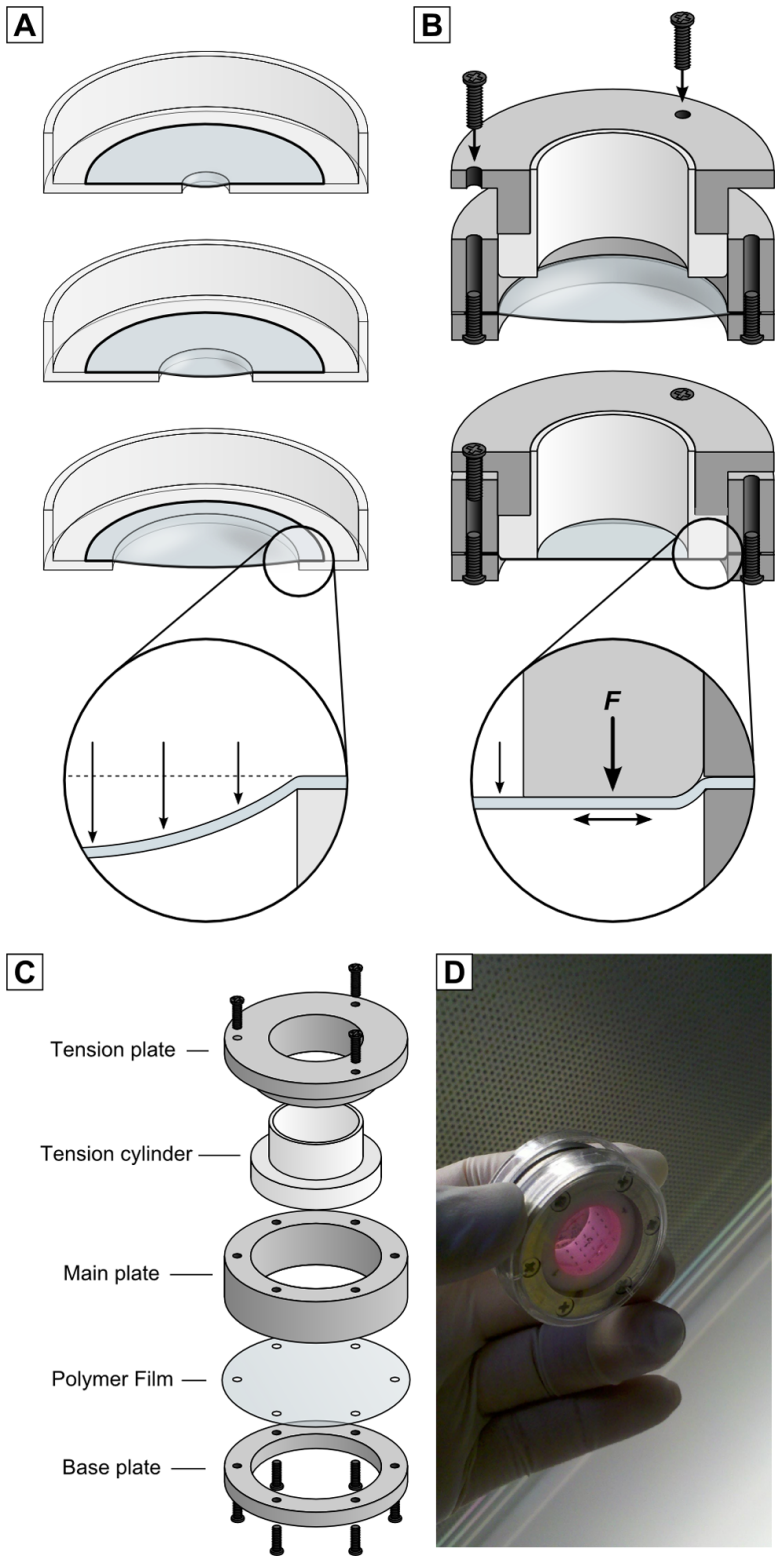
The polymer film holder. (A) Unsupported polymer films bow under the weight of culture media limiting the imaging area. (B) The polymer film holder applies tension to flatten the film. (C) The polymer film holder is comprised of a tension plate, tension cylinder, main plate and base plate. (D) The assembled film holder during cell culture.

Aclar film offers advantages for EM sample preparation and is compatible with HPF. Current CLEM protocols using HPF require the use of a specialised microscope stage which increases the free working distance of the objective lens and limits optical imaging to a disk smaller than 1.3 mm in diameter [15], [17]. Conventional fixation procedures enable the use of larger pieces of Aclar, but due to the pliability of the film it must be supported by a glass coverslip [12], [16], [18]. In addition to increasing the free working distance of the objective lens, combining Aclar with a coverslip can introduce additional refractive interfaces between the sample and lens which can cause image aberrations [23]. We attempted to perform the optical imaging without the glass coverslip support, but found that gluing Aclar film over a hole drilled into tissue culture plasticware does not support the weight of tissue culture medium and results in a curved film which restricts optical imaging (Figure 1A). To overcome this problem we designed a film holder which flattens the polymer film by keeping it under tension (Figure 1B). The film holder was modelled on the dimensions of a 35 mm culture dish (Figure S1) to be compatible with a wide range of inverted microscopes. The film holder produces a flat surface for imaging through a tensioning mechanism, whereby the film is clamped between a base and main plate (Figure 1C) and force is applied laterally through a tension plate and cylinder to flatten a relatively large (16.5 mm diameter) area for imaging. The film tension can be adjusted with the top screws. The holder is mainly constructed of stainless steel with the exception of the PTFE (polytetrafluoroethylene) tension cylinder, which provides a suitable contact surface for tissue culture. The large area flattened by the film holder enables multiple regions to be individually extracted and processed for simultaneous CLEM experiments. Importantly, the design also enables the user to cut out discs from the film using a punching device to directly follow up the optical imaging with cryofixation by HPF.

### Assessment of polymer film substrates for CLEM

**Figure 2:**
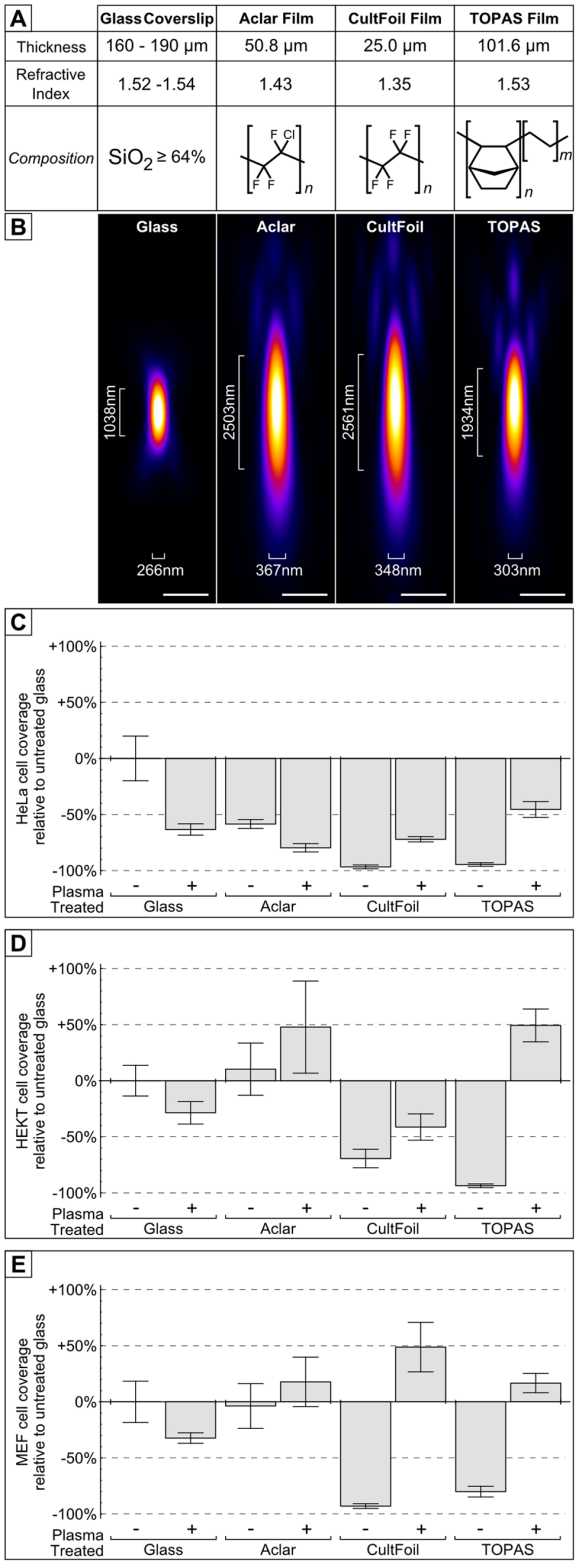
Comparison of polymer film CLEM substrates. (A) Three polymer films (Aclar, CultFoil and TOPAS) were investigated as potential substrates for CLEM. (B) Green PS-Speck microspheres (λ_ex_505 nm/ λ_em_515 nm, 0.175 µm Φ) were dried onto the surface of a glass coverslips, Aclar, CultFoil and TOPAS, then mowiol mounted onto microscope slides. Optical z-stacks were acquired of individual microspheres on each substrate, then combined using Huygens PSF distiller to calculate the PSF for each substrate. The resolution (Full width at half maximum) of each PSF was characterised using the MetroloJ plugin for ImageJ. (C) HeLa cells (5×10^4^), (D) HEKT cells (5×10^4^), or (E) MEF cells (2.5×10^4^) were seeded into 12-well plates containing glass coverslips, Aclar, CultFoil and TOPAS, with or without 30 s argon glow discharge. The cells were cultured for 24 hours before being rinsed to remove non-adherent cells. The cells were fixed and stained with toluidine blue before acquiring images, which were quantified using ImageJ to provide a measurement of cell area coverage relative to the untreated glass coverslip. (Images shown in Figure S2; Scalebars: 1 µm).

Objective lenses are typically optimized for the refractive index and thickness of a glass coverslip, which differ from the properties of polymer films like Aclar. To select an optimal polymer-based substrate for optical imaging we tested the optical characteristics of different polymer films (Figure 2), including Aclar film (polychlorotrifluoroethylene; PCTFE), CultFoil (polytetrafluoroethylene; PTFE), and TOPAS (cyclic olefin copolymer; COC). To characterise the optical properties of each film, we recorded and analysed empirical point spread functions (PSFs) collected through each substrate (Figure 2B). We acquired 3D optical data of sub-diffraction sized (0.175 µm Φ) green fluorescent microspheres on the surface of each polymer film and a glass coverslip, then generated a PSF for each surface using Huygens PSF distiller. The Full-Width-at-Half-Maximum (FWHM) value of each PSF was analysed using the MetroloJ package of ImageJ to provide a measure of their practical resolution limit. Each polymer film exhibited spherical aberration artifacts, consistent with their deviation from the standard thickness and refractive index of a glass coverslip. The refractive index and thickness of TOPAS was the closest match to the properties of glass coverslips and the resolution achieved with TOPAS was superior to both Aclar and CultFoil.

To determine the suitability of polymer films for mammalian cell culture, samples of glass coverslips, Aclar, CultFoil, or TOPAS were exposed to glow-discharge plasma [26] and seeded with HeLa, HEKT or MEF cells (Figure 2C–E; Figure S2). After 24 hours of cell culture, the morphology and coverage of each cell line was evaluated and quantified relative to the coverage of cells grown on untreated glass coverslips. Glow-discharge treatment reduced cell attachment to glass coverslips in all three cell lines, but improved cell attachment to most of the polymer films. Cell attachment to CultFoil and TOPAS was significantly improved by glow-discharge treatment, which enabled cells to attach without forming aggregates (Figure S2). In comparison, Aclar film showed only moderate improvements for HEKT and MEF cell attachment after glow-discharge treatment. The results indicate that each polymer film is a viable substrate for mammalian tissue culture. Considering the better optical properties of TOPAS, the results collectively show that glow-discharged TOPAS is the optimal substrate for live-cell CLEM.

**Figure 3:**
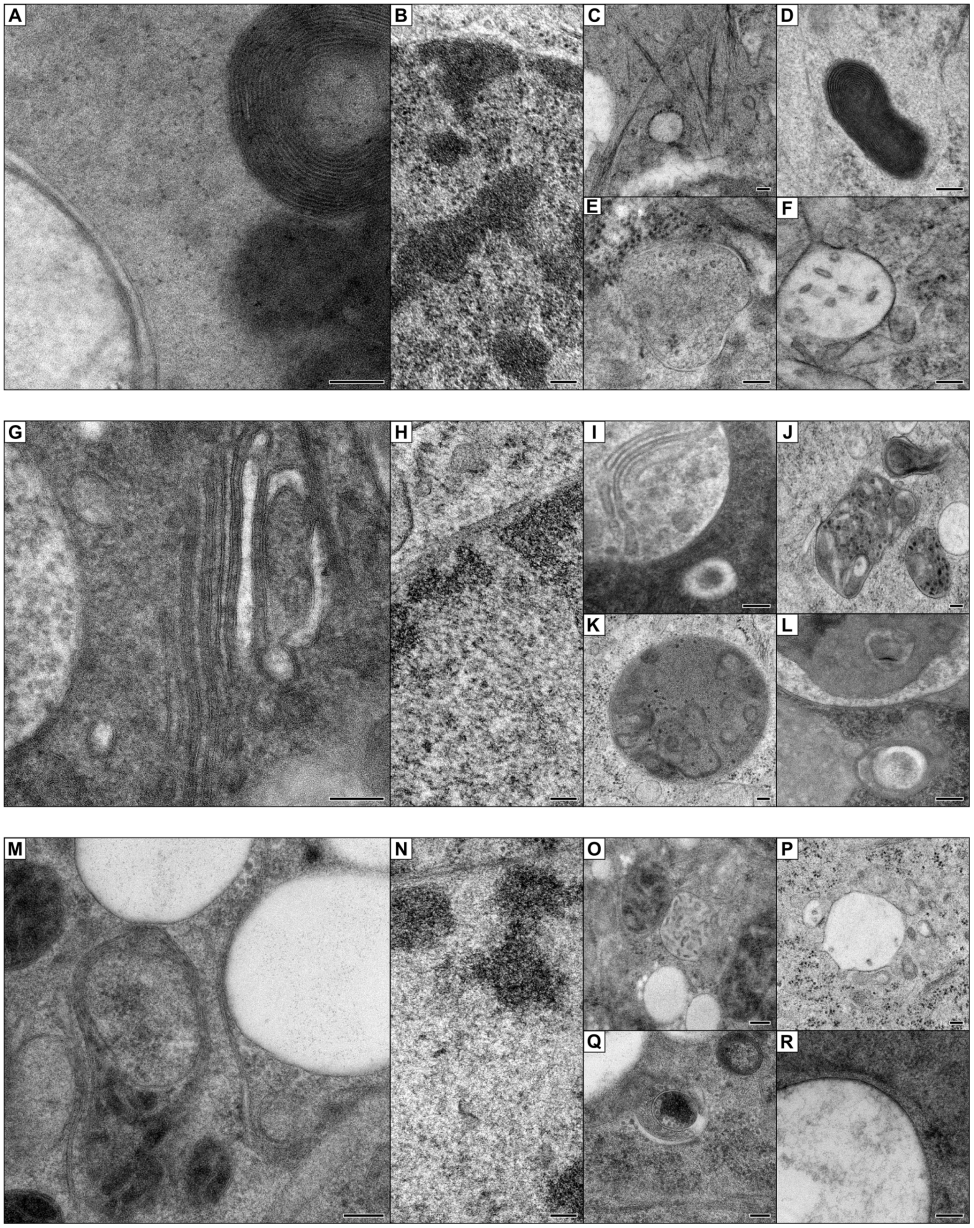
High pressure freezing of polymer film substrates. HEPG2 cells cultured on discs of (A–F) Aclar, (G–L) CultFoil, and (M–L) TOPAS were high pressure frozen and freeze substituted for ultrastructural characterisation. To assess the quality of cryopreservation, (A,G,M) multi-layered membrane structures were examined for membrane continuity, and (B,H,N) nuclei were checked for evidence of ice crystal damage. (C–F, I–L, O–R) The ultrastructural preservation of membrane vesicles was also examined. (Scalebars: 100 nm).

We next tested the suitability of CultFoil and TOPAS for cryo-fixation by HPF. Aclar is currently the only polymer film substrate widely used for HPF [14], [15], [17], [19], [21]. HEPG2 cells were seeded onto pieces of glow-discharged Aclar, CultFoil and TOPAS. After 24 hours of cell culture the polymer films with the cell monolayer were dipped in cryoprotectant, then frozen using a Leica EM PACT2 (Figure 3). We used 20% BSA in DMEM as primary cryoprotectant since the alternative cryoprotectant 1-hexadecene caused the TOPAS film to disintegrate. After freeze-substitution, resin embedding and ultramicrotomy, ultrathin sections were stained for TEM imaging. TOPAS film did not readily detach from the resin block and was therefore sectioned *in situ*, whereas the Aclar and CultFoil films freely detached from the resin before sectioning. Similar preservation quality was observed for each of the polymer films. These results indicate that both CultFoil and TOPAS are viable substrates for HPF.

### A multiscalar reference system for spatial alignment

**Figure 4:**
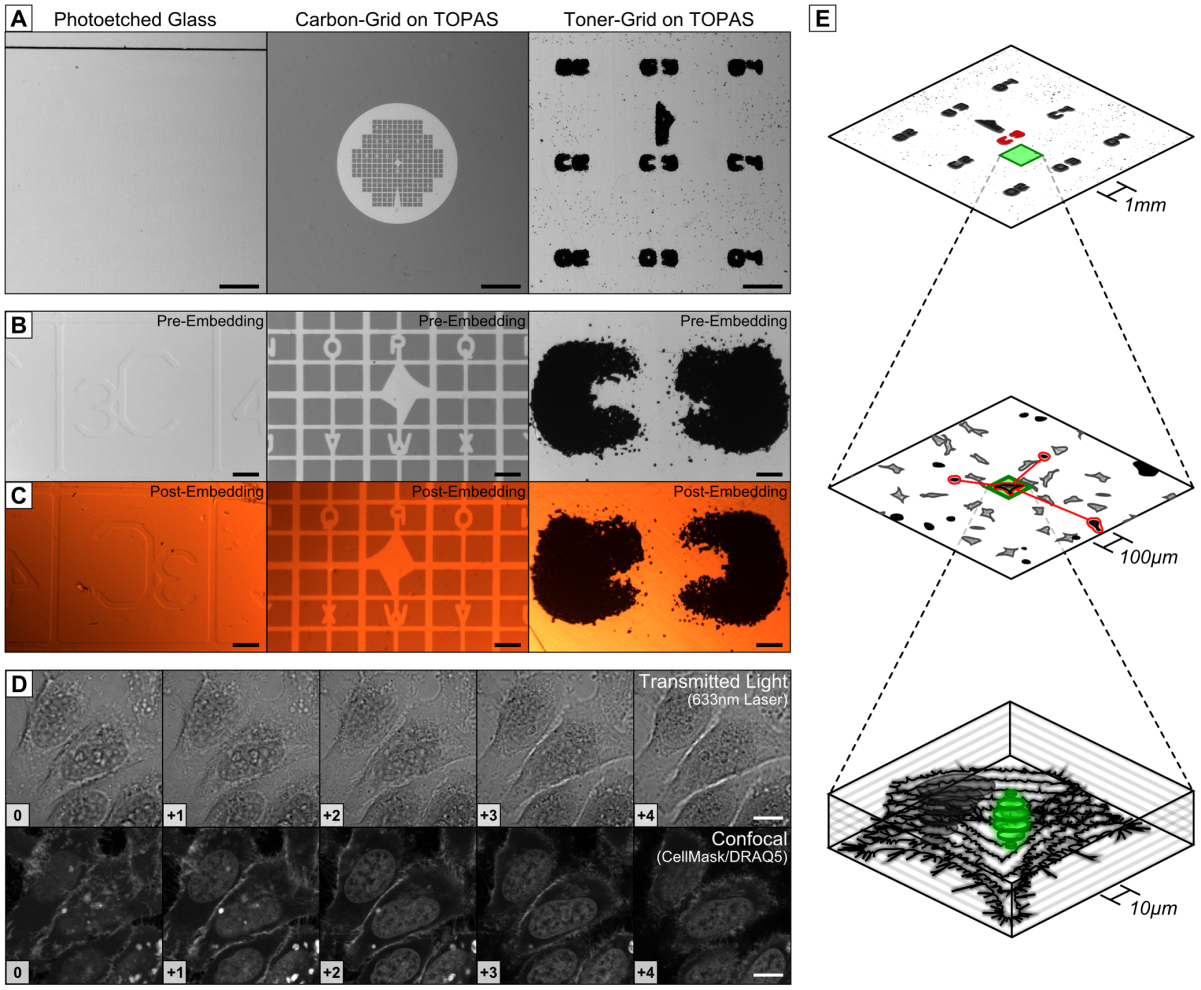
The procedure for subcellular spatial alignment offers improved visibility and tracking of a region of interest. (A–C) The visibility and legibility of glass photo-etched coverslips (left), carbon-based grids (middle) and toner-based grids (right) on TOPAS film were compared at (A) low magnification, (B) high magnification and (C) after resin embedding. (D) Z-stack of HeLa cells stained with CellMask Deep-Red and DRAQ5 imaged by transmitted light and CLSM fluorescence, with the relative position of optical sections indicated as an integer (bottom-left, 0.96 µm per section). (E) The multiscalar fiducial marker system allows for hierarchical spatial alignment. A reference grid allows relocation of a ROI within the sample, toner particles serve as fiducial markers for relocating a cell within the ROI, and intrinsic cellular features allow relocation of a subcellular target. (Scalebars: A = 1mm; B,C  = 100 µm; D = 10 µm).

We next aimed to establish a reliable strategy for aligning subcellular structures using a simple reference grid in conjunction with extrinsic and intrinsic fiducial markers (Figure 4E). We used a toner transfer technique [24], [25] to produce highly visible toner-based alphanumeric reference grids on TOPAS (Figure 4A–C). In contrast to other methods for producing reference grids (Figure 4 left and middle column) [11], [12], the toner-based grids are legible without the aid of a microscope (see also Figure 1D). Viewing at higher magnifications reveals the presence of individual toner particles which can serve as more precise extrinsic fiducial markers (Figure 4B) and which remain visible after resin embedding and trimming (Figure 4C). Toner-based reference grids provide spatial reference points on two different scales, allowing a region-of-interest (ROI) to be mapped relative to the grid coordinates and subsequently relative to individual toner particles.

Relocating a subcellular target often relies on aligning EM images with morphological features identified using phase contrast or differential interference contrast (DIC) microscopy [17], [27]–[30], which are not related to the image contrast of TEM or fluorescence microscopy. Instead we used the fluorescent dyes DRAQ5 and CellMask Deep-Red to label the cell nucleus and plasma membrane respectively, which are key morphological features in EM images (Figure 4D). DRAQ5 and CellMask fluoresce at the far-red end of the spectrum, are compatible with live-cell imaging and remain fluorescent after PFA fixation. Far-red dyes minimise the risk of phototoxicity during live cell imaging [31], and dye visibility in fixed cells enables morphological disturbances to be probed after fixation. Optical sectioning of cells stained with DRAQ5 and CellMask provides valuable 3D information which can be used to guide ultramicrotomy to the correct Z-plane, particularly after restorative deconvolution of the data set which can improve image resolution by a factor of 2 laterally and a factor of 4 axially [32]. The use of DRAQ5 and CellMask in combination with restorative deconvolution therefore provides an ideal source for intrinsic fiducial markers for aligning TEM images to their corresponding position in the cell.

The toner-based grids, toner-particles and the intrinsic cellular features collectively form a multiscalar fiducial marker system that facilitates spatial alignment in all three dimensions (Figure 4E). At the lowest magnifications, the alphanumeric toner grid enables the excision of one or more ROIs from the film using a scalpel or punch for TEM processing without using a microscope. At intermediate magnifications, the toner particles within the selected ROI help to relocate the target cell for ultramicrotomy [33]. The information retrieved from the 3D-confocal fluorescence data using intrinsic cellular features guides ultramicrotomy to the correct depth, which is determined during sectioning by recording the thickness of each ultrathin section. The thickness of an ultrathin section can be measured with a high degree of precision [34], [35], but we have found section interference colour to be an adequate estimate of section thickness since the error margin is still several orders of magnitude smaller than the axial resolution of a confocal microscope [36], [37].

### Validation of the CLEM procedures

**Figure 5:**
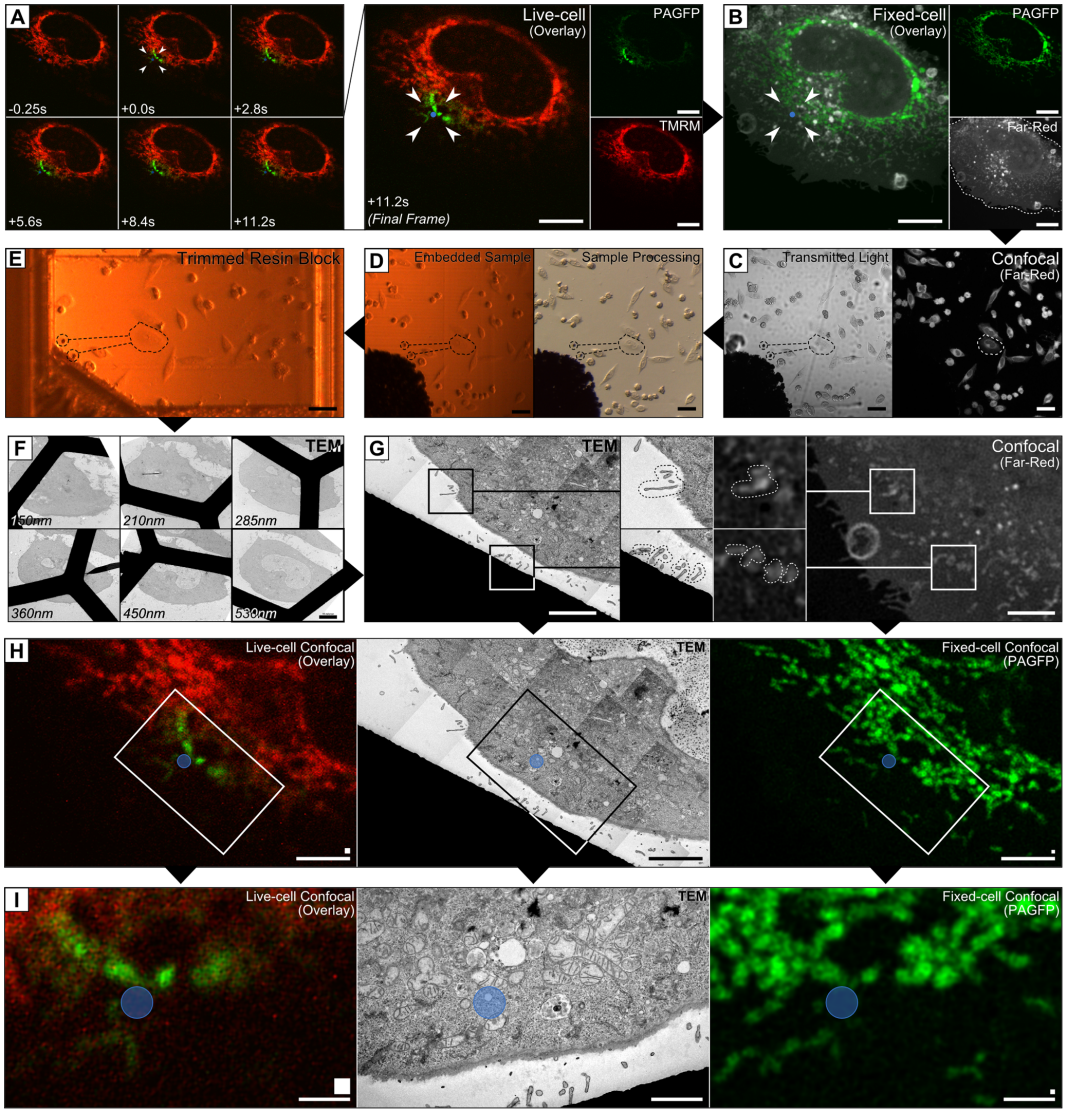
Application of the procedure to investigate mitochondrial photodamage. (A) HeLa cells on toner-gridded TOPAS were transfected with PAGFP-Mito and stained with TMRM, CellMask Deep-Red and DRAQ5 before live-cell imaging. A 1.2 µm diameter region (arrowheads) within a cell was photo-irradiated prior to fixation. (B) After reactivating the PAGFP, the cell was optically sectioned and (C) neighbouring toner particles were imaged to define the position of the cell (dashed black lines). (D) The target region was cut from the TOPAS by scalpel for processing and resin embedding, using the reference grid to guide extraction. (E) Toner particles were used to guide targeted trimming of the resin block, which was then sectioned (F) to the depth identified from the optical data. (G) The TEM data was spatially aligned using intrinsic cellular features identified by optical imaging (dotted lines in enlarged insets). (H–I) The spatially aligned data is shown with the photoirradiated region indicated by a blue circle. (Scalebars: A,B,F  = 10 µm; C,D,E  = 50 µm; G,H  = 5 µm; I = 2 µm. Original pixel sizes are indicated by squares above the scalebars: H = 10×10 pixels; I = 5×5 pixels).

To verify our live-cell CLEM workflow we investigated the EM morphology of individual mitochondria after their selective depolarisation with laser light. High-intensity 488 nm laser light results in mitochondrial injury and irreversible depolarisation [38], [39]. We sought to determine whether mitochondrial photodamage coincided with acute changes in mitochondrial ultrastructure. HeLa cells cultured on TOPAS film were transfected with mitochondrially-targeted photoactivatable GFP (PAGPF-Mito), then stained with TMRM (Tetramethylrhodamine Methyl Ester) to follow mitochondrial depolarisation [40], and with DRAQ5 and CellMask Deep-Red to provide fiducial markers. Time-series fluorescence data was collected before and after photoirradiation of a target cell (Figure 5A). The cells were chemically fixed 12 seconds after photoirradiation by adding double strength fixative directly to the culture media. After fixation all cellular PAGFP was photo-activated and optical sections of the green and far-red fluorescence channels were acquired at high resolution (Figure 5B) to provide the intrinsic fiducials for spatial alignment. Low magnification images (DIC and fluorescence) were acquired to determine the nearest grid point and define the position of the cell relative to toner particles (Figure 5C). After acquisition of the optical images a scalpel was used to excise the region of TOPAS film containing the target cell for TEM processing and resin embedding (Figure 5D). Toner particles present on the TOPAS film were used to guide trimming for targeted ultramicrotomy (Figure 5E). Without removing the TOPAS from the resin block, the film was trimmed by sectioning (150 nm thick) with a glass knife until the first evidence of resin, after which 75 nm ultrathin sections were cut to a target depth (320 nm to 960 nm) determined from the optical data (Figure 5F). TEM grids were selected for imaging based on the recorded thickness of each section, providing a measure of its 3D location in the resin block. Grids were imaged by TEM to collect a high-resolution montage of the target cell, which was then aligned with the nucleus and filopodia observed in the deconvolved optical data (Figure 5G). As demonstrated by the spatially aligned data (Figure 5H–I) our procedure enabled the relocation of an intracellular ROI with a high degree of precision. Formaldehyde fixation alone does not sufficiently preserve the cells for electron microscopy, but the high diffusion speed of formaldehyde enables rapid uniform immobilisation of the cell monolayer and subsequent high resolution optical imaging. Minimising the interim time between live-cell imaging and fixation effectively enables correlation between the live-cell confocal and TEM images (Figure 5I), provided that the live-cell data is of sufficiently high resolution and can first be correlated with the fixed-cell optical data.

**Figure 6:**
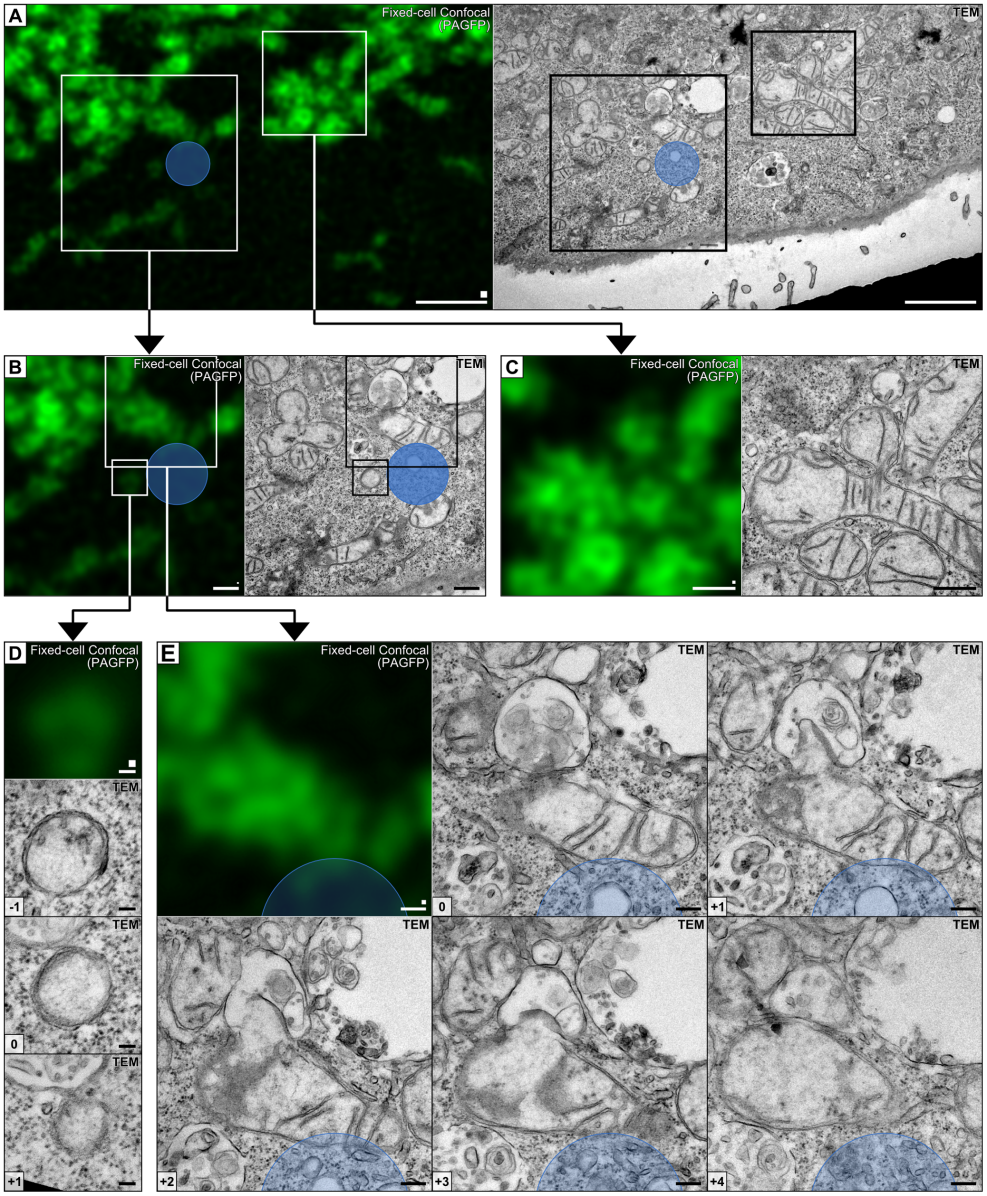
High-magnification of the spatially aligned region. (A) The spatially aligned data showing the photoirradiated region indicated by a blue circle, with insets of (B) mitochondria near the photoirradiated region and (C) mitochondria away from the photoirradiated region. (D–E) High-magnification insets of mitochondria within 1µm of the photoirradiation region with TEM images acquired of adjacent sections. The position of adjacent sections relative to the aligned reference section is indicated as an integer (bottom-left). (Scalebars: A = 2 µm; B,C  = 1 µm; D = 100 nm; E = 200 nm. Original pixel sizes are indicated by squares above the scalebars: A = 5×5 pixels; B–E  = 1×1 pixel).

Using the spatially aligned data (Figure 6A) we sought to investigate the ultrastructure of mitochondria in the photo-irradiated region. TEM imaging of the target region revealed two PAGFP-positive structures in the direct vicinity of the photo-irradiated region (Figure 6B). The smaller PAGFP-positive vesicle-like structure had a diameter of 400 nm and was surrounded by a double membrane but lacked internal membranes (Figure 6D). The presence of PAGFP and the double membrane indicated a mitochondrial origin. The other PAGFP positive structure (Figure 6E) displayed morphology more typical for mitochondria (Figure 6C). Interestingly, images of adjacent ultrathin sections revealed interactions with an adjacent endo-lysosomal vacuole, and the mitochondrial membrane was found to form an invagination into the vacuolar lumen. The physiological significance of this observation has yet to be determined and warrants further investigation. Given that equivalent observations are not possible with live-cell CLSM or TEM alone the results collectively validate our live-cell CLEM procedure.

## Discussion

CLEM techniques have great potential to facilitate discoveries in all areas of cellular biology. However, CLEM remains technically challenging due to the difficulty of relocating a region of interest in 2D and 3D with adequate precision. Our procedure offers a way to track a region of interest over three orders of magnitude. Using toner particles the region of interest can easily be viewed by eye and low magnification allowing the tracking of the same position during imaging, embedding and targeted ultramicrotomy. The use of polymer films as direct imaging substrate offers additional advantages, including the ability to extract and separately process several regions of interest in close proximity, and fewer refractive index changes due to omission of the glass coverslip. In addition the ability to perform HPF fixation offers an advantage in cases where a larger sample area needs to be screened in optical imaging before HPF, and the timing of the cryo-fixation step is less important [22], [41]. The current procedure for combining HPF with CLEM limits the cell culture area to a 1.3 mm diameter disc of Aclar or sapphire [9], [14], [15], [17], but enables rapid freezing of the sample within 5–10 seconds of optical imaging. Using our procedure for HPF of living tissue after optical imaging requires a 1.2–1.5 mm plunger biopsy punch and a circular cutting surface of PTFE or brass (25 mm diameter); The film holder is placed on top of the cutting surface, the punch is pressed into the desired region, and the punch plunger is used to deposit the disk into a HPF membrane carrier. This process takes three to four times longer than the current HPF-CLEM configuration, however, the mechanical processes involved are highly amenable to automation. Further development of this strategy could therefore reduce this time considerably.

Our CLEM procedure relies on standard optical and electron microscopy equipment, with the only required specialized equipment being the film holder. We have provided a schematic for the holder so that it can be reproduced in a workshop (Figure S1). We have used a single film holder to conduct a range of CLEM experiments including a recent study investigating the fate of mitochondria during Parkin-mediated mitophagy [42]. One of the key advantages of the film holder is the ability to use any suitable film as a CLEM substrate enabling the further improvement and optimisation of substrates for microscopic applications. TOPAS offered the best optical properties of the tested films when using lenses optimised for glass coverslips due to its thickness and refractive index. However, the optical properties of CultFoil and Aclar are in part offset by the strength and chemical resistance of CultFoil and the usefulness of Aclar as a substrate for cryofixation by HPF. We found that although TOPAS was more challenging to use in HPF due to its solubility in the commonly used cryoprotectant 1-hexadecene, it is possible to perform HPF with TOPAS if BSA is used as the primary cryoprotectant (Figure 3). Given the diverse range of modern polymers currently available, it is likely that more viable CLEM substrates will emerge in the near future.

The use of highly visible toner-based grids for CLEM is a central advantage of our procedure. The toner transfer technique enables the large-scale production of custom reference grids (Figure S3) using commonly available equipment. Beyond the use of conventional toner more advanced results could be achieved by modifying the toner composition or surface functionalization for CLEM investigations into tissue engineering and bioprinting [43]–[45]. In addition it should be possible to produce tissue culture dishes using our toner-based grid system on polymer substrates commercially. This would eliminate the need for the polymer film holder and further facilitate the uptake of our procedure.

The acquisition of 3D optical data is often overlooked due to the use of transillumination techniques such as DIC and phase contrast microscopy [17], [27], [29], [30]. Confocal microscopy is a more viable strategy for recording the 3D distribution of intrinsic fiducial markers. Some caution should be taken when directly aligning 3D optical with EM data as image artifacts can misrepresent the true position of intrinsic fiducial markers. Image processing techniques such as optical deconvolution [46] and EM distortion correction [47]–[49] are therefore important for improving the quality and accuracy of CLEM. In addition it is also important to consider inaccuracies that occur during ultramicrotomy as ultrathin sections are often not cut perfectly parallel to the confocal optical sections. For example, if two structures separated by 10 µm are at the same depth, an ultramicrotome misalignment of just 0.4° relative to the optical sections will cause them to appear in different ultrathin sections. It is therefore important to use intrinsic features in direct proximity of the target region during the subcellular correlation (as shown in Figure 5G). Intrinsic fiducial markers may also be used for computational based spatial alignment strategies which currently rely on Quantum Dots or fluorescent microbeads [50]. This strategy uses fiducial marker coordinates to calculate a transformation matrix, which is then used to translate other coordinates between the different imaging techniques. An alignment deviation below 100 nm can be achieved by using a large number of reference fiducials to calculate the optimal 2D transformation matrix [50], [51], however it is important to note that this precision value relates to lateral but not axial alignment due to the use of a 2D transformation matrix. While this strategy is viable for spatially aligning images of sections [50]–[54], the computational alignment of volumes inevitably requires the calculation of 3D transformation matrices.

The toner based reference system might be a critical step in facilitating higher throughput of samples for CLEM. Augmented reality (AR) is a computer vision technique that uses fiducial markers to align and superimpose virtual objects into a real-world environment [55]. The high contrast of our toner based reference system would be suited for such an application. Considering the present availability of open-source AR engines [56]–[58] and the diverse range of consumer electronics already using AR [59]–[62], the technology could conceivably be adopted for automating CLEM spatial alignment. AR would also be used in CLEM strategies that employ focussed ion beam milling with scanning EM [63] as toner particles would be visible by SEM imaging after ablation of the film [64]. Cyclic olefin copolymers including TOPAS are increasingly being used to fabricate lab-on-chip devices [65]–[67] and further functionalization could open up new opportunities for improving CLEM. Combining CLEM with lab-on-chip technologies and computer vision may therefore be a valuable strategy for the development of high-throughput CLEM.

Our results demonstrate a simple CLEM procedure with considerable scope for development. Simple procedures based on widely accessible materials and equipment are more readily adapted to suit new applications. By eliminating the use of non-standard equipment, we have developed a CLEM procedure that is readily accessible for implementation and further development.

## Materials and Methods

### Materials

For live-cell microscopy experiments TMRM and CellMask Deep-Red from Invitrogen, and DRAQ5 from Biostatus were used. Aclar (50.8 µm PCTFE; ProSciTech), Cultfoil (25 µm PTFE; Zeiss) and TOPAS 6013 (101.6 µm COC; TOPAS Advanced Polymers). All other reagents were obtained from Sigma-Aldrich.

### Cell Culture

MEF cells were a gift from Sharon Tooze (London Research Institute) [68]; HeLa (ATCC CCL-2) and HEKT (ATCC CRL-11268) cells were gifts from David Jans (Monash University); and HEPG2 (ATCC HB-8065) cells were a gift from Matthew Watt (Monash University). All cell lines were cultured in DMEM containing 10% FCS. HeLa cells were transiently transfected with Mito-PAGFP (Addgene) 24 hours prior to the experiment using Lipofectamine 2000 (Invitrogen) according to the manufacturer's protocol.

### Characterisation of Polymer Substrates for CLEM

Aclar (50.8 µm PCTFE; ProSciTech), Cultfoil (25 µm PTFE; Zeiss) and TOPAS 6013 (101.6 µm COC; TOPAS Advanced Polymers) were assessed for CLEM suitability. To assess the optical properties of the films, PS-Speck Green microbeads (λ_ex_505 nm/ λ_em_515 nm) were dried onto the surface of Aclar, CultFoil, TOPAS and a glass coverslip. The substrates were Mowiol mounted onto slides before the acquisition of optical z-stacks on each substrate using an Olympus FluoView FV1000 inverted confocal microscope, with a 60× water immersion objective lens (PLAPO, NA 1.00; Olympus). The z-stacks were collected at a resolution of 25 nm per x-y pixel and 160 nm per section, with the condenser aperture set to 140 µm. The microbeads were excited by 488 nm laser and fluorescence emission was recorded at 500 nm ±50 nm by photomultiplier tube, using the Olympus Fluoview acquisition software (version 1.7c). The acquired data was imported into Huygens PSF distiller (Huygens professional 64bit, version 4.2.1p8), which automatically aligned and combined 5 data sets from each sample into a corresponding 16-bit PSF tiff stack. The distilled stacks were imported into ImageJ (version 1.45 s) and analysed using the MetroloJ plugin (release version) to characterise the imaging resolution. The example PSF images were prepared by rescaling the distilled PSF images with bicubic interpolation, saturating 0.35% of the pixels and converting to a “Fire” look-up-table to improve visibility.

To compare cell adhesion to the films, 5×10^4^ HeLa cells were seeded into the wells of a 12-well plate containing Aclar, CultFoil, and TOPAS, either without surface treatment or after a 30 s glow discharge in an Argon atmosphere (0.5 torr) [69]. Non-adherent cells were rinsed away after 24 hours of cell culture, before PFA fixation, Toluidine blue staining, and Mowiol embedding for imaging. The cells were imaged on an Olympus BX60 upright using a 20× dry objective (UPlanApo, NA 0.70; Olympus), with a brightfield ColorView II FireWire camera controlled by the AnalySIS LS Professional (Soft Imaging Solutions, version 2.6) acquisition software. The red and green channels of each colour image were added together and thresholded to produce a binary image of cell outlines. The binary image was analysed using the ImageJ “Analyse Particles” function to determine the cell area coverage in each field-of-view.

To assess the suitability of the polymer films for high pressure freezing, discs of Aclar, Cultfoil and TOPAS 6013 were cut using a 1.2 mm Harris Uni-Core tissue punch (ProSciTech), then individually heat-welded to the surface of a 6 cm tissue culture dish plastic using a soldering iron. The dishes were sterilised using 80% ethanol and UV light before being seeded with HEPG2 cells. After 24 hours of cell culture, the discs were retrieved and frozen by HPF using a Leica EM PACT2 with RTS by dipping each disc in cryoprotectant (20% BSA in DMEM) and loading it into a 200 µm membrane carrier (1.5 mm diameter; Leica). The membrane carrier was briefly dipped in 1-Hexadecene immediately prior to freezing. Frozen samples were transferred to a Leica AFS unit for freeze substitution for 50 hours (24 hours at −90°C, 6 hour transition, 8 hours at −60°C, 12 hour transition to 0°C) using a fixative substitution media cocktail (1% osmium tetroxide, 0.2% uranyl acetate, and 3% water in acetone). The samples were rinsed three times with anhydrous acetone at room temperature, then infiltrated with a graduated series of Procure-Araldite in acetone (25%, 50%, 75%, 100%, 100%) and polymerised for 48 hours at 60°C. The membrane carriers were removed using a razor blade, and the samples were sectioned (70–80 nm) using an Ultracut UCT ultramicrotome (Leica).

### The toner transfer technique for preparation of CLEM grids

**Figure 7:**
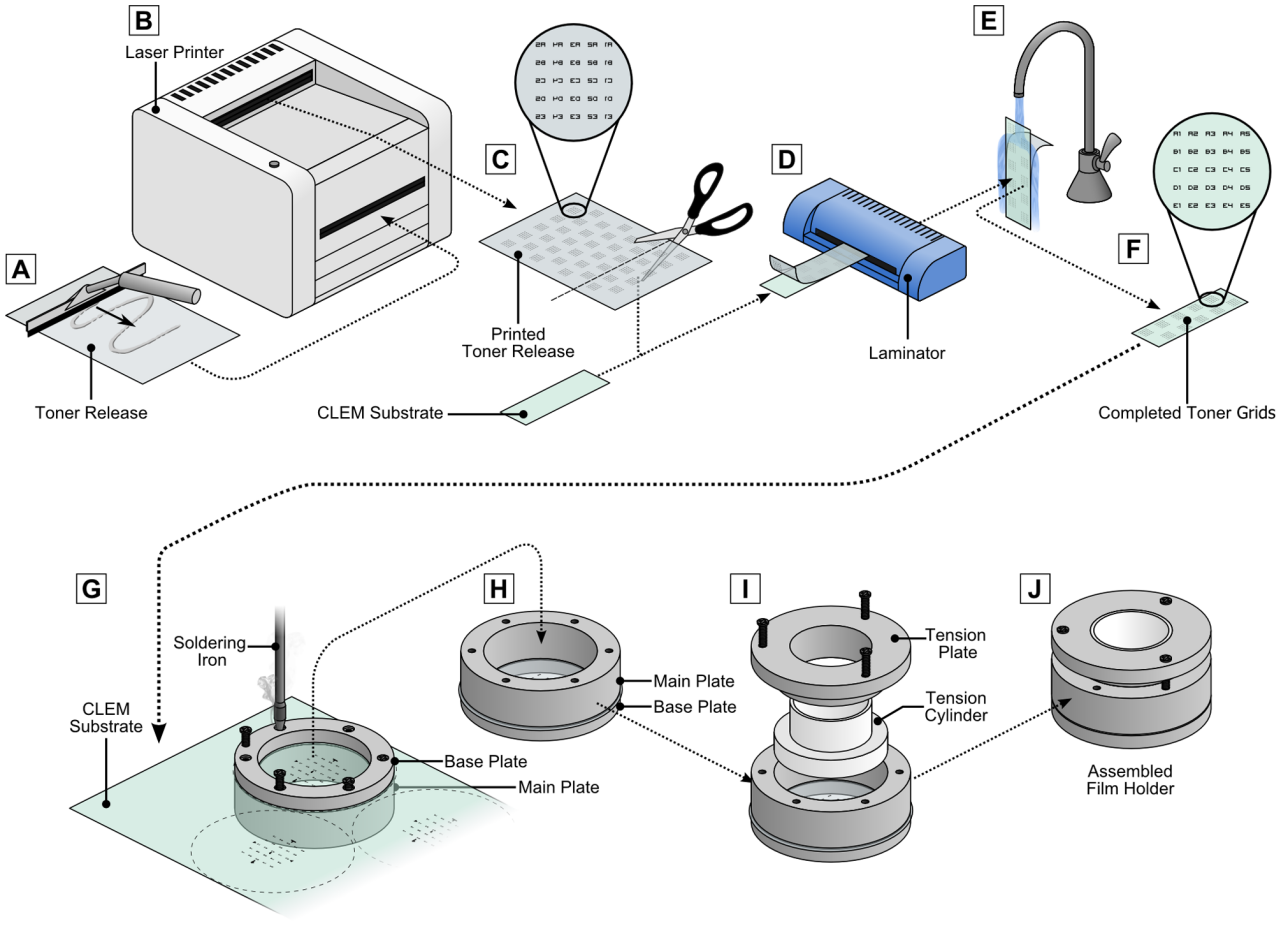
Procedure for the preparation of toner based grids and assembly of the film holder. (A–F) The toner transfer technique uses a water soluble toner-release sheet to transfer toner to the CLEM substrate. (A) A toner-release sheet is prepared by coating a laser printer projector transparency with 10% PVA and left to dry. (B) Grid patterns are designed using vector graphics software, then horizontally flipped before being printing on the PVA side of the toner-release sheet. (C) The printed toner-release is cut into strips matching the CLEM substrate. (D) The PVA side of the toner-release is pressed against the CLEM substrate, then run through a laminator until sufficient adhesion is achieved. (E) The substrate is separated from the toner-release using running water to dissolve the PVA, or by soaking overnight. (F) The toner-marked CLEM substrate can be cut into suitable sizes for cell culture. (G–J) Toner marked CLEM substrates are mounted into the film holder prior to cell culture. (G) The CLEM substrate is placed up-side-down between the base plate and main plate of the film holder and a soldering iron is used to melt holes into the CLEM substrate, allowing the six base screws to be fitted. (H,I) The film holder is turned right-side-up, and the tension plate and tension cylinder are installed into the central cavity of the holder. (J) After tightening the three tensioning-screws, the toner-marked CLEM substrate and film holder are sterilised with 80% ethanol and UV light before cell culture.

Toner grids were prepared using a Xerox Phaser 6360 laser printer and a generic office laminator (Lowell) (Figure 7A–F). The grids were designed using the open-source SVG editor Inkscape [70]. Carbon masked reference grids were also prepared as outlined by Jiménez et al. [12] and gridded coverslips acquired from MatTek for comparison with the toner grids. Successful implementation of the toner transfer technique required printing onto an intermediate surface, the “toner release”, from which the toner was transferred onto the polymer film. (Printing directly to the CLEM substrate caused damage of the film through the mechanical and thermal stress of printing process.) Our procedure used a water-soluble toner release, which was prepared by coating an A4 (210×297 mm) overhead projector transparency (3 M) with a solution of 10% polyvinyl alcohol (PVA), which was then left to dry on a level surface. Once the PVA had dried, mirror images of the grid patterns were printed onto its surface using a laser printer. The printed toner release sheets can be stored if kept in a dry dust-free environment. The CLEM substrate was prepared for toner deposition by exposure to glow-discharge plasma to improve toner adhesion [71]. To perform the transfer, similarly sized pieces of the toner release and CLEM substrate were pressed together then fed into a pre-warmed office laminator, using temperatures between 90°C and 130°C to melt the toner [72]. Best results were achieved by passing the films through the laminator multiple times. Finally, running water was used to dissolve the PVA of the toner release, leaving the toner attached to the CLEM substrate. Non-adherent toner was washed from the substrate before storage. Each toner grid was loaded into a film holder prior to cell culture (Figure 7G–J). The base plate and main plate of the film holder were first assembled up-side-down on either side of the CLEM substrate, which was also up-side-down. Screw holes were then prepared in the CLEM substrate by pressing a hot soldering iron into each screw hole of the base plate. The film was clamped using the six base screws. After inserting the tension plate and tension cylinder into the central cavity of the holder, the film was flattened by tightening the three top tensioning screws. The complete assembly of the film holder was sterilised with 80% ethanol and UV light before cell culture.

### Intrinsic Fiducial markers

The cell nucleus and digitations on the plasma membrane were used as intrinsic fiducial markers in the optical data. The plasma membrane and nucleus were stained 30 minutes prior to imaging using 2.5 µg.ml^−1^ CellMask Deep-Red and 2.5 µM DRAQ5 respectively.

### CLEM experiment

HeLa cells were cultured for 24 hours on a gridded CLEM substrate restrained within the universal film holder, which had been sterilised with 80% ethanol and UV light. The cells were transfected with PAGFP-Mito for 24 hours before replacement of the culture media with DMEM+10%FCS without phenol red. The cells were stained with 500 nM TMRM, 2.5 µg.ml^−1^ CellMask Deep-Red and 2.5 µM DRAQ5 30 minutes prior to imaging via live-cell confocal microscopy on an Olympus FluoView FV1000 inverted confocal microscope, using a 60× objective lens (PLAPO, NA 1.20; Olympus). A circular region (1.2 µm in diameter) within a target cell was micro-irradiated for 250 ms using 405 nm and 488 nm lasers with post-objective powers of 287 µW and 967 µW respectively. Live cell time-series data was acquired for 10 seconds immediately after micro-irradiation, followed by the direct addition of 8% PFA/0.1M PB equal to the volume of culture media. To aid sample navigation, a montage of images was acquired of the surrounding cells and toner particles. All PAGFP-mito was then reactivated (by scanning with the 405 nm laser at 645 µW for 500 ms), and three-dimensional image data was acquired of the CellMask, DRAQ5 and PAGFP-Mito with an lateral pixel sampling of 34 nm/pixel and an axial pixel sampling of 320 nm per section, with the condenser aperture set to 140 µm. The optical data was deconvolved using Huygens Professional deconvolution software (Huygens professional 64bit, version 4.2.1p8) for 40 iterations using a calculated PSF with Maximum Likelihood Estimation and a signal-to-noise of 20. The theoretical resolution of the deconvolved optical data was calculated by using the same parameters to deconvolve an empirical PSF measured on TOPAS to provide a re-estimation of the PSF after deconvolution [32]. The FWHM resolution of the re-estimated empirical PSF was calculated to be 225 nm laterally and 1599 nm axially using the MetroloJ plugin for ImageJ.

The sample was post-fixed for 1 hour with 2.5% glutaraldehyde in 0.1M PB, then processed for transmission electron microscopy (TEM) using a BioWave Pro microwave system (Pelco) for conventional microwave assisted processing. Samples were further fixed in 1% aqueous osmium tetroxide, en-bloc stained with 2% aqueous uranyl acetate, dehydrated in ethanol (50%, 70%, 90%, 100%, 100%) and acetone (100%, 100%), infiltrated with Procure-Araldite in acetone (25%, 50%, 75%, 100%, 100%), and polymerised for 48 hours at 60°C. The position of the target cell was tracked throughout sample processing using a Leica M125 dissection microscope with the IC80 HD camera (Leica). The region of interest was located before ultramicrotomy using a low magnification confocal montage. After trimming away the TOPAS layer on an Leica Ultracut UCT ultramicrotome, the specimen feed was set to 70 nm and ultrathin sections were cut. The thickness of each section was recorded (n = 26; mean thickness: 74.8 nm ±2.2 nm; estimated total depth: 1870 nm) based on interference colour [34], [35] to provide an estimate of its physical depth in the sample. Grids containing the sections closest to the target z-position (∼640 nm above basal surface) were stained (First 11 sections; estimated thicknesses: 75 nm, 75 nm, 60 nm, 75 nm, 75 nm, 90 nm, 80 nm, 70 nm, 75 nm, 80 nm, 75 nm; estimated depths: 0 nm to 830 nm) with uranyl acetate for 5 minutes and lead citrate for 3 minutes. TEM imaging was conducted at 80 kV on a Hitachi H-7500 TEM using a Gatan 791 MultiScan side mount CCD camera and DigitalMicrograph (Version 1.71.38) acquisition software. The target cell was relocated using the confocal image montage for navigation, and the deconvolved confocal data was used to confirm initial estimates of the z-position. Filopodia near the photoirradiation ROI were used to identify the correct section (7^th^ section; estimated thickness: 80 nm; estimated depth: 530 nm), and a TEM montage of 25 images was manually acquired at 10,000× magnification. Image lens distortion was corrected using the Distortion Correction plugin for FIJI (FIJI Is Just ImageJ, Version 1.47 g), and the images were manually aligned into a single montage image using GIMP (GNU Image Manipulation Program, Version 2.8.2). The distortion corrected TEM montage was aligned with the deconvolved optical data (2^nd^ optical section above the basal surface) using filopodia, the nucleus, and other intrinsic features as anchor points. All subsequent TEM data was aligned directly to the distortion corrected TEM montage. Correlated fluorescence data was obtained by scaling and aligning TEM images to the TEM montage, extracting the aligned region from the fluorescence channels, then performing the reverse operations with bicubic interpolation.

### Software

Deconvolution and PSF acquisition were performed using Huygens Professional [73], and PSF resolution analysis was conducted using the MetroloJ [74] plugin for ImageJ [75]. The toner grids were designed using the open-source SVG editor Inkscape version 0.48 [70]. TEM montage data was corrected using the Distortion Correction [47] plugin for FIJI [76]. The TEM montage and final image correlation were performed manually using the GNU Image Manipulation Program [77].

## Supporting Information

Figure S1 **Technical schematic for manufacture of the universal film holder.** The universal film holder is comprised of the (A) tension plate, (B) tension cylinder, (C) main plate and (D) base plate. The tension plate, main plate and base plate are each constructed of stainless steel rod, and the tension cylinder is constructed of PTFE rod, all of which are turned by lathe. The M2 screws and threading of the main plate can be substituted for any screw that remains flush on the countersunk base plate.
(TIFF)

Figure S2 **Cell attachment to polymer film substrates.** (A) HeLa cells (5×10^4^), (B) HEKT cells (5×10^4^), or (C) MEF cells (2.5×10^4^) were seeded into 12-well plates containing glass coverslips, Aclar, CultFoil and TOPAS, with or without 30 s argon glow discharge pre-treatment. The cells were cultured for 24 hours before being rinsed to remove non-adherent cells. The cells were fixed and stained with toluidine blue before acquiring images. (Scalebars: 50 µm).
(TIFF)

Figure S3 **Toner grid design examples for use with the film holder.** (A) An example of the toner grid pattern used for the CLEM experiment in Figure 5 is shown. (B) A magnified and un-flipped view of the Cartesian grid used in the design, and the resulting toner pattern after printing directly to a projector transparency. (C) A magnified and un-flipped view of an alternatively designed Hex grid, and the resulting toner pattern after printing directly to a projector transparency. (Scalebars: B,C  = 1 mm).
(TIFF)
