# Supplementary figures and images for: An Improved Procedure for Subcellular Spatial Alignment during Live-Cell CLEM
Source: PLoS One. 2014 Apr 22;9(4):e95967. doi: 10.1371/journal.pone.0095967 (PMC3995996; doi:10.1371/journal.pone.0095967)

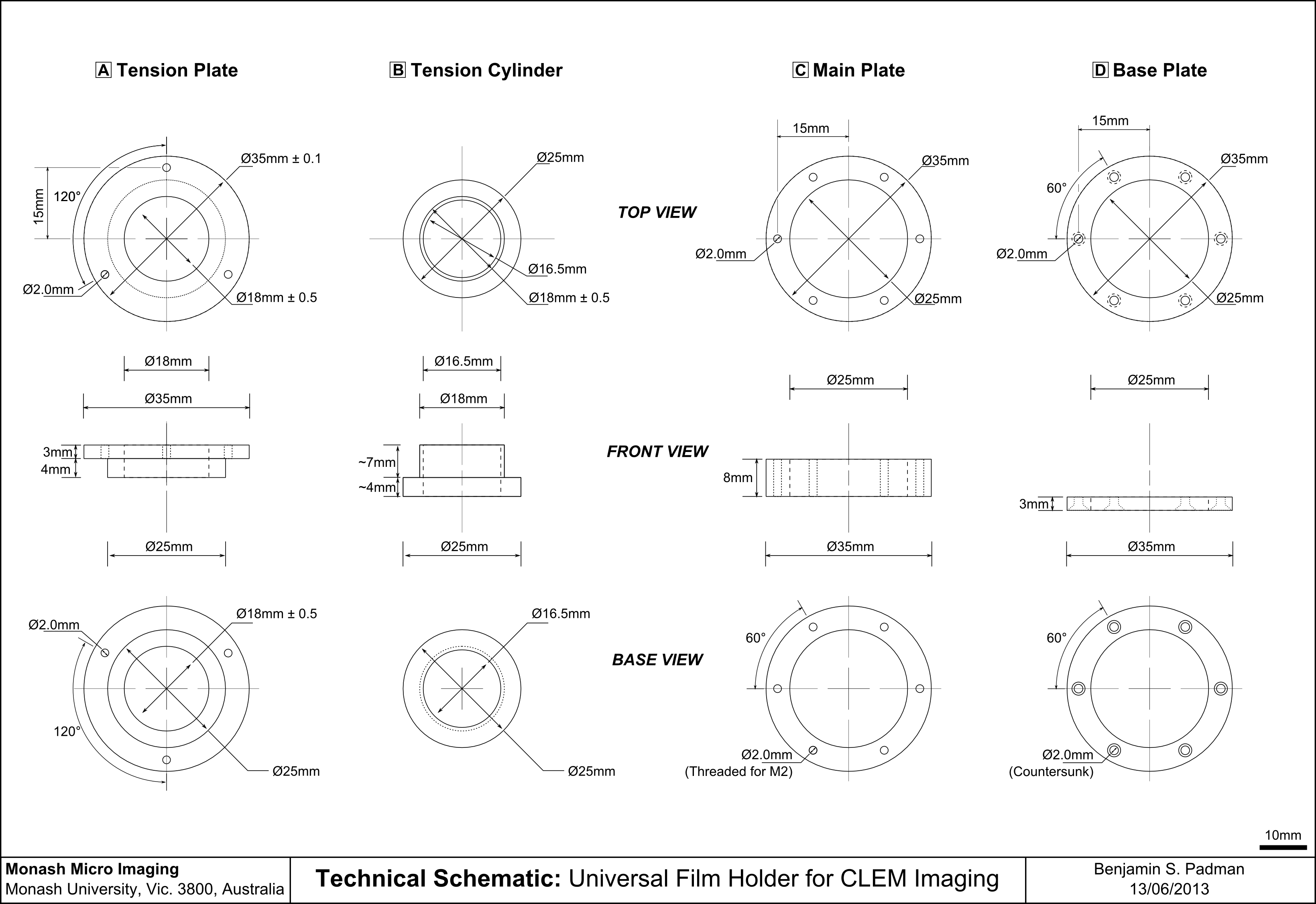

Supplement: Figure S1 — Technical schematic for manufacture of the universal film holder. The universal film holder is comprised of the (A) tension plate, (B) tension cylinder, (C) main plate and (D) base plate. The tension plate, main plate and base plate are each constructed of stainless steel rod, and the tension cylinder is constructed of PTFE rod, all of which are turned by lathe. The M2 screws and threading of the main plate can be substituted for any screw that remains flush on the countersunk base plate. (TIFF) [file pone.0095967.s001.tiff]

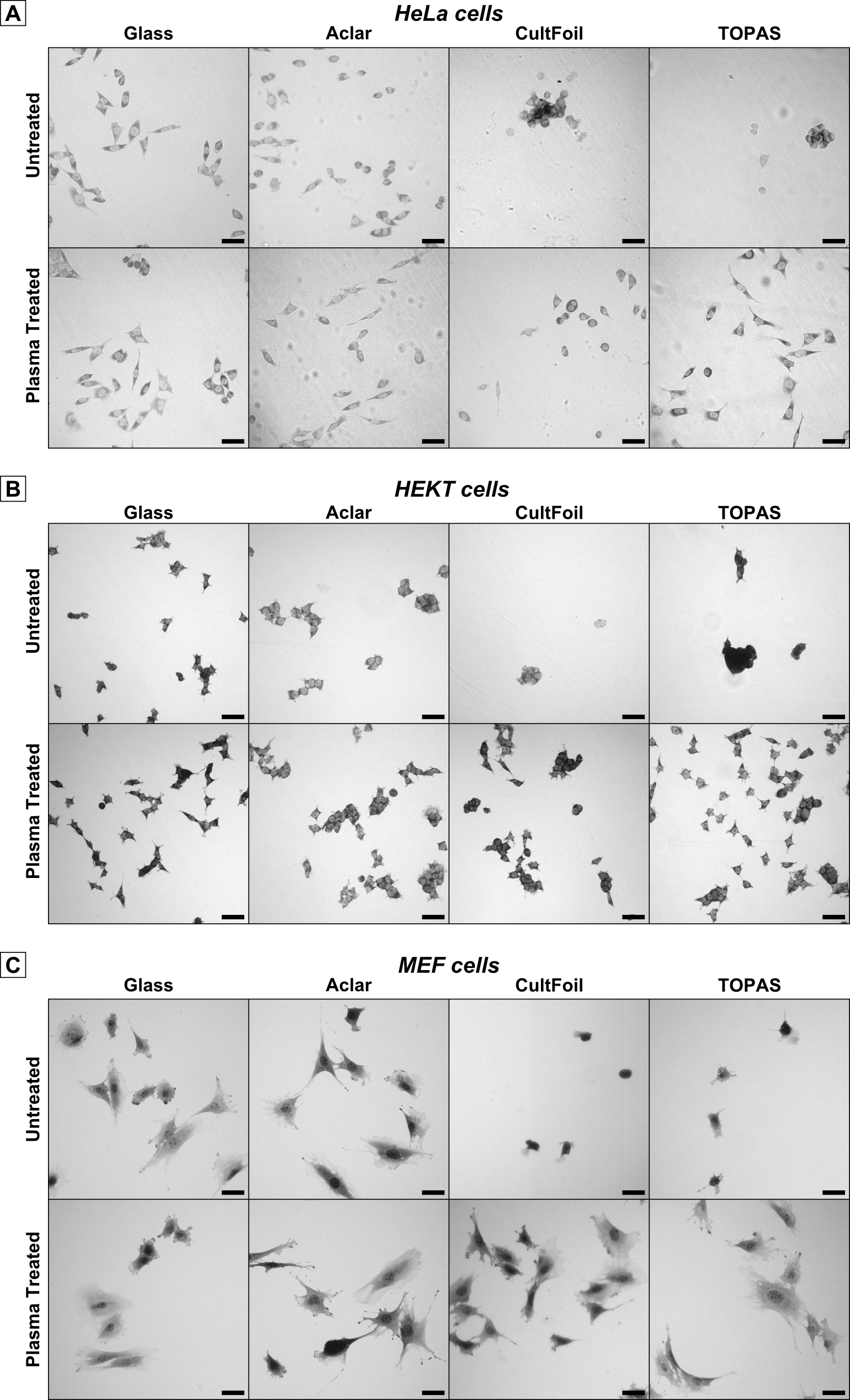

Supplement: Figure S2 — Cell attachment to polymer film substrates. (A) HeLa cells (5×10^4^), (B) HEKT cells (5×10^4^), or (C) MEF cells (2.5×10^4^) were seeded into 12-well plates containing glass coverslips, Aclar, CultFoil and TOPAS, with or without 30 s argon glow discharge pre-treatment. The cells were cultured for 24 hours before being rinsed to remove non-adherent cells. The cells were fixed and stained with toluidine blue before acquiring images. (Scalebars: 50 µm). (TIFF) [file pone.0095967.s002.tiff]

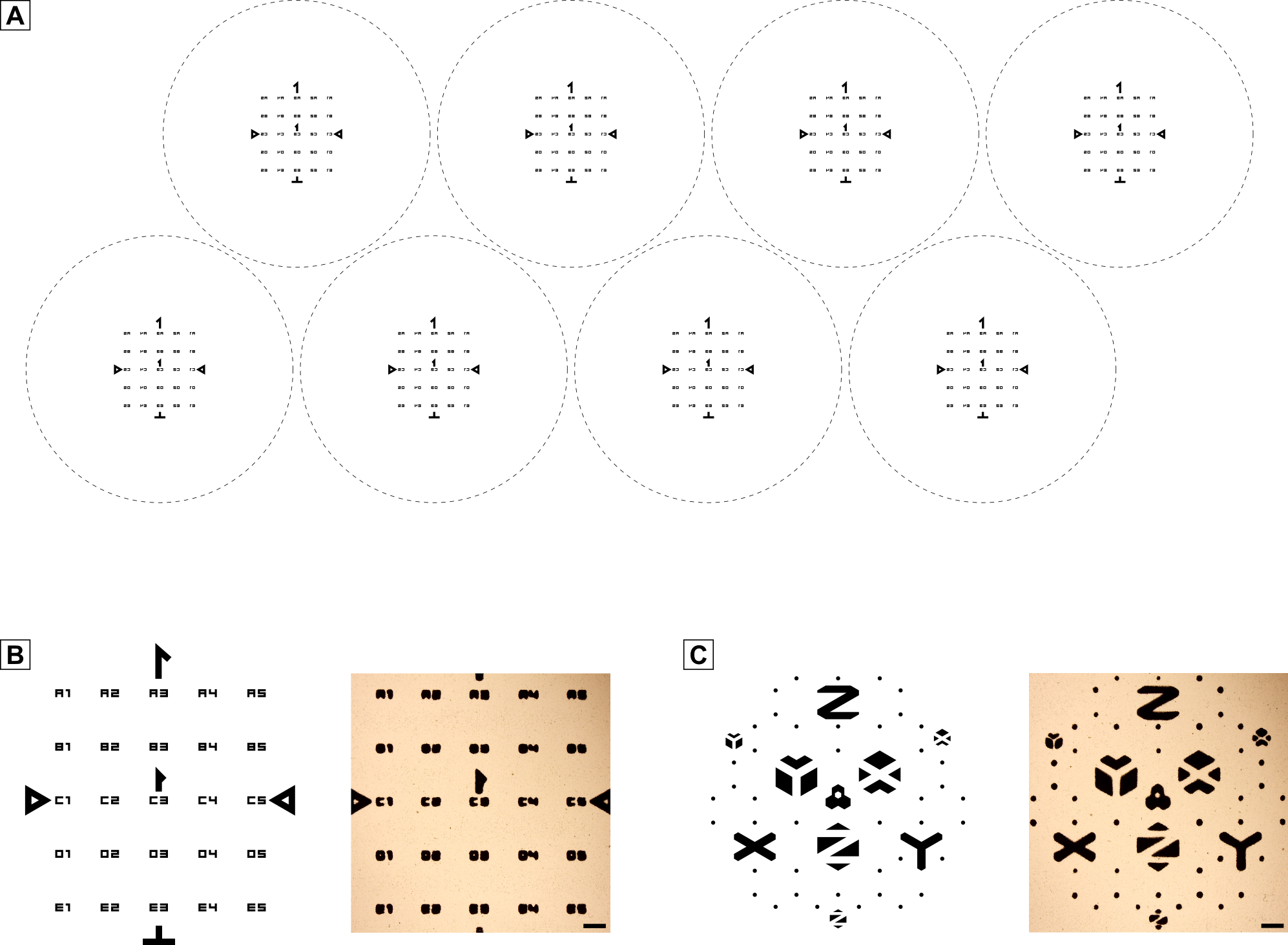

Supplement: Figure S3 — Toner grid design examples for use with the film holder. (A) An example of the toner grid pattern used for the CLEM experiment in Figure 5 is shown. (B) A magnified and un-flipped view of the Cartesian grid used in the design, and the resulting toner pattern after printing directly to a projector transparency. (C) A magnified and un-flipped view of an alternatively designed Hex grid, and the resulting toner pattern after printing directly to a projector transparency. (Scalebars: B,C = 1 mm). (TIFF) [file pone.0095967.s003.tiff]
